# Nurse-led motivational interviewing to change the lifestyle of patients with type 2 diabetes (MILD-project): protocol for a cluster, randomized, controlled trial on implementing lifestyle recommendations

**DOI:** 10.1186/1472-6963-9-19

**Published:** 2009-01-30

**Authors:** Renate Jansink, Jozé Braspenning, Trudy van der Weijden, Louis Niessen, Glyn Elwyn, Richard Grol

**Affiliations:** 1 Scientific Institute for Quality of Healthcare, Radboud University Nijmegen Medical Centre, P.O. box 9101, 6500 HB Nijmegen, The Netherlands; 2 Department of General Practice, School for Primary Care and Public Health, Maastricht University, P.O. Box 616, 6200 MD Maastricht, The Netherlands; 3 Department of International Health, Johns Hopkins School of Public Health, 615 N. Wolfe Street, Ste. E1002 Baltimore, MD 21205, USA; 4 School of Medicine, Health Policy and Practice, University of East Anglia, Norwich, NR4 7TJ, UK; 5 Department of Primary Care and Public Health, School of Medicine, Cardiff University, Heath Park, Cardiff, CF14 4XN, UK

## Abstract

**Background:**

The diabetes of many patients is managed in general practice; healthcare providers aim to promote healthful behaviors, such as healthful diet, adequate physical activity, and smoking cessation. These measures may decrease insulin resistance, improve glycemic control, lipid abnormalities, and hypertension. They may also prevent cardiovascular disease and complications of diabetes. However, professionals do not adhere optimally to guidelines for lifestyle counseling. Motivational interviewing to change the lifestyle of patients with type 2 diabetes is intended to improve diabetes care in accordance with the national guidelines for lifestyle counseling. Primary care nurses will be trained in motivational interviewing embedded in structured care in general practice. The aim of this paper is to describe the design and methods of a study evaluating the effects of the nurses' training on patient outcomes.

**Methods/Design:**

A cluster, randomized, controlled trial involving 70 general practices (35 practices in the intervention arm and 35 in the control arm) starting in March 2007. A total of 700 patients with type 2 diabetes will be recruited. The patients in the intervention arm will receive care from the primary care nurse, who will receive training in an implementation strategy with motivational interviewing as the core component. Other components of this strategy will be adaptation of the diabetes protocol to local circumstances, introduction of a social map for lifestyle support, and educational and supportive tools for sustaining motivational interviewing. The control arm will be encouraged to maintain usual care. The effect measures will be the care process, metabolic parameters (glycosylated hemoglobin, blood pressure and lipids), lifestyle (diet, physical activity, smoking, and alcohol), health-related quality of life, and patients' willingness to change behaviors. The measurements will take place at baseline and after 14 months.

**Discussion:**

Applying motivational interviewing for patients with diabetes in primary care has been studied, but to our knowledge, no other study has yet evaluated the implementation and sustainability of motivating and involving patients in day-to-day diabetes care in general practice. If this intervention proves to be effective and cost-effective, large-scale implementation of this nurse-oriented intervention will be considered and anticipated.

**Trial registration:**

Current Controlled Trials ISRCTN68707773.

## Background

Professionals' performance in lifestyle counseling is suboptimal, yet it is very important that healthcare providers promote healthful behaviors for patients with type 2 diabetes[1,2]. There are studies indicating that healthful diet and physical activity decrease insulin resistance and improve glycemic control, lipid abnormalities, and hypertension, thereby lowering the risk of cardiovascular disease (CVD) [3-6]. Smoking is known to be particularly dangerous; it doubles the risk of CVD for those with and without diabetes[7]. Lifestyle counseling requires more focused support for professionals so that they can adequately support their patients[8,9].

With the increasing prevalence of diabetes due to aging and the increasing average weight in the population, the problems of lifestyle counseling are becoming more urgent[9]. Diabetes mellitus is a major cause of morbidity and mortality worldwide. About half a million people are known to have diabetes in the Netherlands, and this number is expected to increase by 36% in the next 20 years[10]. The age- and sex-adjusted prevalence of type 2 diabetes is 2.9%; 3.1% for women and 2.7% for men[11]. Patients aged more than 70 years account for almost 50% of all patients with type 2 diabetes [11].

Effective diabetes care is based on two elements: structured care and a patient-centered approach [12-14]. These elements lead to improvements of patient outcomes and process outcomes, and they play an important role in lifestyle counseling for diabetes patients [15-17]. In the Netherlands, diabetes care is provided mainly in primary care (80–90%), and in most practices, a primary care nurse has the tasks of providing lifestyle counseling in a structured manner and involving patients in managing their disease[18,19]. Nevertheless, some studies suggest that many healthcare providers, nurses included, lack the skills to promote lifestyle change [20-24]. A practical tool such as the patient-oriented counseling technique of motivational interviewing (MI) can contribute to implementing the lifestyle recommendations[20,25]. Controlled trials in general practices have shown that MI is an effective strategy in the treatment of various diseases [26-30]. However, just training primary care nurses in MI will not be sufficient; it is important to embed MI in an implementation strategy[13].

There is a wide range of implementation strategies aimed at improving the provision of diabetes care in primary care. Multifaceted professional interventions (such as counseling, auditing, and feedback) and organizational interventions (such as revision of professional roles, changes in medical record systems, and arrangements for follow-up) that facilitate the structured and regular review of patients have proven to be effective in improving care[13]. However, very few studies have focused on integrating some of these implementation strategies effectively into professional behavior in daily work in general practice.

Our Motivational Interviewing to Change the Lifestyle of Patients with Type 2 Diabetes (MILD) Study is intended to improve type 2 diabetes care in accordance with the national guidelines for lifestyle counseling by having primary care nurses, who will be trained in lifestyle MI and who will implement structured care in general practice. The impact of the implementation strategy will be evaluated in various ways. First, the effect of the implementation strategy on the nurses' care and the relevant patient outcomes will be examined and compared with those of usual care. Second, there will be a process evaluation of the exposure of the implementation strategy and its feasibility. Third, since this study will determine both the effects and the costs of the implementation strategy, we plan to evaluate it economically to establish the cost-effectiveness. The aim of this study protocol is to describe the design and methods of a study to evaluate the effects, costs, process, and cost-effectiveness of an implementation strategy for motivating and involving patients in lifestyle issues as a part of their diabetes management.

Our research questions will be:

1. What is the effect of an implementation strategy aimed at the routine MI by primary care nurses compared to usual care with regard to:

(a) The care process

(b) The metabolic parameters, such as glycosylated hemoglobin (HbA1_c_), blood pressure, and lipids

(c) Lifestyle changes; diet, exercise, smoking, and alcohol consumption

(d) Health-related quality of life

(e) Patients' willingness to change behaviors? [Effect evaluation]

2. To what extent do primary care nurses take part in the implementation strategy, and is the strategy feasible in the view of patients and nurses? [Process evaluation]

3. What is the incremental cost-effectiveness ratio of our implementation strategy compared with that of the usual care of a primary care nurse? [Economic evaluation]

## Methods/Design

### Study design

The study is a cluster, randomized, controlled trial involving 70 general practices and 700 patients with type 2 diabetes in the Netherlands. Each practice is staffed with a primary care nurse who will be allocated to either:

- The control arm of practices in which patients with diabetes receive only usual care from the primary care nurse.

- The intervention arm of practices in which patients with diabetes receive care from nurses who will be skilled in MI and authorized to use it.

We will include all appointments for each patient (the guidelines require four appointments), so that each patient will be followed for 14 months.

### Ethical approval and informed consent

The Medical Ethics Committee of the University of Nijmegen has granted ethical approval. The trial is registered as ISRCTN68707773[31]. The general practitioner (GP) and the research team will send a letter of information about the project to each eligible patient. The privacy of the participating patients will be protected, and all data will be coded and processed anonymously. It will be made clear in the informed consent form that each patient can stop his of her participation in the study at any moment without any consequence for the quality of his or her usual diabetes care. The patient will be asked to sign the informed consent form and return it to Nijmegen University to allow further contact regarding the research.

### Recruitment of general practices and patients

**Figure 1:**
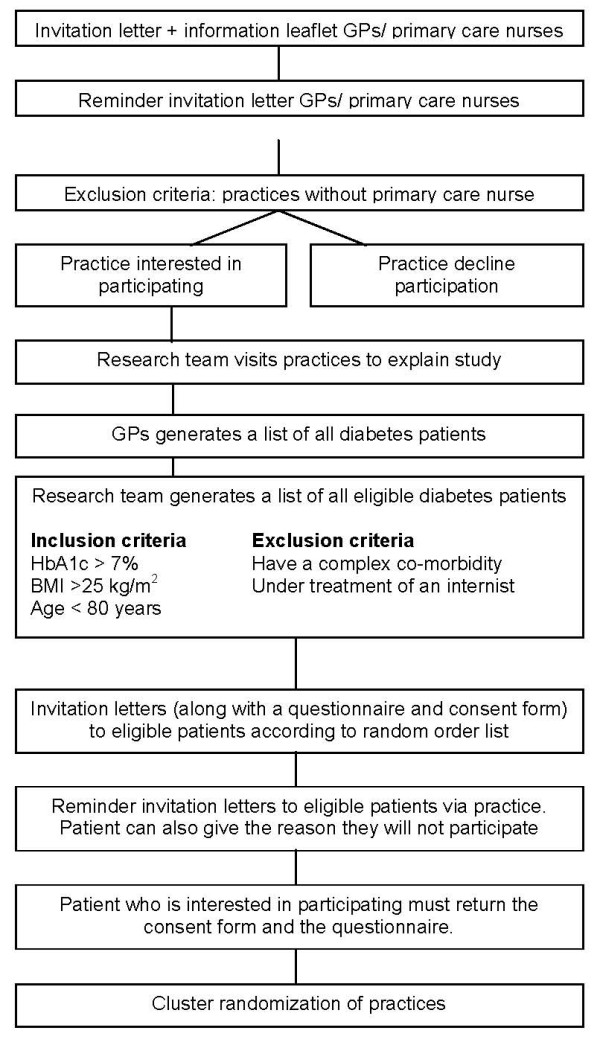
**Flowchart of the process for recruiting general practices and patients with diabetes**.

The trial will be carried out with general practices and their patients with diabetes. Figure 1 shows the pathway with which general practices and patients will be recruited.

#### General practices

Practices in the south of the Netherlands will receive a letter of invitation with an information leaflet about the study. General practices that do not employ a primary care nurse will be excluded from the study. A member of the research team (RJ) will visit all practices that express their intention to participate. At least one GP and the primary care nurse of a practice willing to participate will be present during the appointment. We will explain the study in detail and provide them with a full information package describing the aims, methods, and expected outcomes of the study. Nonresponders will be reminded by letter 4 weeks later. Invitation letters will be sent in several rounds until we find 70 general practices that will participate.

#### Patients

Patients with type 2 diabetes type will be eligible to participate if they are younger than 80 years, their most recent (frequently no longer than a year ago) HbA1_c _concentration is more than 7.0%, their body mass index is more than 25 kg/m^2^, and they are receiving care from a general practice that employs a primary care nurse. Patients with complex coexisting medical conditions (e.g. mental illness or end-stage cancer) and those being treated by an internist will be excluded. Two members of the research team will make a list of all eligible patients in the participating practices by extracting means data from medical files. Each of these patients will be sent a letter with the GP's invitation to participate in the study. The letter will include information about the design of the study, confidentiality of data, the voluntary character of participation, a questionnaire, and the informed consent form. The patients will be asked to respond by returning the signed informed consent form and the completed questionnaire to the research team. Four weeks later, a reminder will be sent to patients who have not responded. Nonresponders will be asked to give their reasons for not participating, so that we can compare the participating and nonparticipating groups.

### Randomization

General practices will be the unit of randomization. An independent person at Radboud University will centrally randomize the 70 general practices in a randomized block design, after the type of practice and urbanization level have been stratified.

### Sample size

In a regular practice, the proportion of patients with diabetes who have an HbA1_c _concentration greater than 7.0% is about 48%[32]. Lifestyle intervention studies have consistently shown that modest changes in the HbA1_c _concentration can reduce the progression from impaired glucose tolerance to diabetes by about 50%[33]. Therefore, we will aim our program to reduce the proportion from 48% to 24% (50% relative risk reduction). With five patients in each practice, 30 practices in each arm of the study will be needed, if we assume an intercluster correlation of no more than 0.05[34], and set alpha at 0.05 and beta at 0.20.

We also calculated our power on another main outcome; the extent to which patients participate, or are willing to participate, in a program of diet, exercise, and smoking cessation. We predict that the willingness to participate in such a program can at least be tripled on the basis of comparable results of intensive stop-smoking programs in general practice[35]. Just as in smoking cessation studies, we assume that the success of lifestyle counseling in usual care is 5% (relative risk reduction). A power calculation based on the these assumptions shows that we need a total of 68 practices (34 in each arm), with 5 patients in each practice for an implementation strategy based on MI. To answer the research questions, we will take a random sample from the target group; and to take dropout into account, we will include 35 practices in each arm with 10 patients in each practice. Recruitment of 70 practices (700 patients) will be feasible.

### Control group conditions/usual care

The primary care nurses in the control group will not have access to the implementation strategy. They will be offered an opportunity to join the training program at the end of the study. The nurses will be instructed to administer usual care consistent with current diabetes guidelines. These guidelines state that, in usual care, the GP pays attention to complaints, glucose regulation, current cardiovascular risk, and the early identification of complications. Patients without complaints and with good metabolic regulation will be invited to check-ups every 3 months. Once a year, the GP will pay extra attention to specific items noted during the consultations[18].

#### Three-monthly check-ups

At the check-up, the GP will ask about well-being, symptoms that indicate hyperglycemia or hypoglycemia, complications in diet and exercise counseling, and medication. The GP will note the patient's weight. The fasting blood glucose value will also be determined every 3 months[18].

#### Annual check-ups

The annual check-up will be extensive. The GP will ask about eye complications, cardiovascular problems, sexual problems, possible causes of and options for treatment, etc. Furthermore, lifestyle counseling (about smoking, physical activity, and alcohol use), physical examination (body weight, blood pressure, and condition of the feet), chronic complications, and laboratory research will be reviewed. The laboratory work will provide values for the fasting glucose, HbA1_c_, creatinine concentration and clearance, lipid spectrum, and urine albumin/creatinine ratio (or albumin concentration)[18].

### The intervention

In addition to instruction for adhering to the prevailing guidelines for diabetes care as described in usual care, the primary care nurses in the intervention practices will receive information about the various components of the implementation strategy: (1) training in MI, (2) adapting the diabetes protocol to local circumstances, (3) introducing a social map for support of lifestyle change, and (4) practice tools for maintaining the training program (Additional file 1). The "diabetes protocol", "social map" and "MI training" will be components of the training program, which consists of 4 half-day training sessions spread over the 1st half year. Primary care nurses will attend these sessions in groups of 5 to 8 people outside the practice. The "practice tools" will be a component of the follow-up and will start after the training program. The implementation strategy will start in the first training session.

#### Motivational interviewing

Motivational interviewing (MI) will be introduced as a tool for behavior change. The MI counseling technique is patient oriented and suitable for brief office visits, and it can be used to improve patient adherence to diet, exercise, and smoking counseling in daily routine[36]. Since the MI technique plays an important role in our study protocol, we have described it in more detail in Additional file 2.

##### Content

The first step in MI is to set the agenda for the consultation together with the patient[36]. "Agenda setting" will be an issue to keep in the back of your mind from the very beginning of the first interview. The basic question here will be, "What are we going to talk about?" The patient will be encouraged to choose one or more key items in this agenda setting. This will make consultations more structured and will lead to a more concrete action plan. After this, the primary care nurse will assess the patient's current behavior and motivation for change by rating and exploring importance and confidence with respect to the chosen key items. She will do this by reflective listening, summarizing, asking open questions, scaling, supporting self-efficacy, rolling with resistance, expressing empathy, and developing discrepancy. If there is a need and sufficient motivation for change regarding one or more items, the primary care nurse will consult with the patient to select one item as the goal for behavior change.

##### Structure

The MI training will be developed in cooperation with a professional trainer, who will also tutor the complete training program. Activities will include group discussions and role plays on specific skills such as use of the ambivalence, supporting self-efficacy, and reflective listening. Video fragments created by a professional involved with MI will be shown, and some video recordings of primary care nurses will be discussed to learn about good and not-so-good points of lifestyle counseling. After each training session, the primary care nurses will have homework: they will study the theory from the training session and apply this theory to at least two patients with diabetes (cases). The instruction to the primary care nurse will be to write down the two consultations and to take these cases to the next training.

#### Protocol for diabetes care

The Dutch guidelines for type 2 diabetes mellitus give recommendations for diagnostics and management to patients with diabetes[18]. However, some recommendations, such as which tasks are to be delegated to primary care nurses and how long a diabetes consultation should last, will have to be adapted to each practice in a localized protocol. During the training, the differences and agreements of several diabetes protocols from general practices will be discussed with the primary care nurses in order to show that there is more than one way to carry out diabetes care.

#### Social map

Practical information will be needed in the practice, e.g., about local diet and exercise programs, so that patients can learn about these programs from the primary care nurse. They will be encouraged to set up a network for (1) contacting local dieticians to make appointments about referral and treatment plans, and (2) gathering local exercise programs and determining whether local government offices or other parties will fund exercise programs (leading to a social map). During the training, the social map will be discussed, and primary care nurses who already have a social map in the general practice will share their ideas with other primary care nurses who do not have a social map in their general practice.

#### Practice tools

The following educational and supportive tools will be distributed after the training program: a laminated instruction chart with counseling techniques, record keeping information, and recommendations for regular telephone follow-ups to patients with diabetes; a help desk for primary care nurses; and a follow-up meeting for primary care nurses. We will ask the primary care nurses to keep up a record of consultation data, such as the stage of change of the patient with regard to diet, physical activity, smoking, and alcohol. The record keeping, like the instruction chart, will be a useful and practical reminder during consultations. The trainer will advise the primary care nurses to monitor patients every 3 months according to the treatment guidelines, to do MI, and to follow up regularly by telephone, which will be every month for the 1st half year and will probably decrease after that.

The research team will function as a help desk; primary care nurses will be able to call the research team for information, who will follow up with three phone calls to inquire about the development of the primary care nurses' health counseling. The nurses' difficulties with the counseling technique will be mentioned to this team and they will look for solutions. When primary care nurses consider a follow-up meeting (an afternoon) important, this meeting will take p[lace. These supportive tools will be used in the follow-up period for maintenance of the MI training.

### Effect evaluation

The aim of the effect evaluation will be to determine whether the implementation strategy has achieved the intended effects on the care process and the patients' clinical outcomes. The research question will be, "What are the effects of an implementation strategy aimed at improving MI skills of primary care nurses compared to the effects of usual care on the care process, the metabolic parameters (such as HbA1_c_, blood pressure, and lipids), lifestyle (diet, exercise, smoking and alcohol consumption), health-related quality of life, and patients' willingness to change their behavior?"

#### Variables and measures

**Figure 2:**
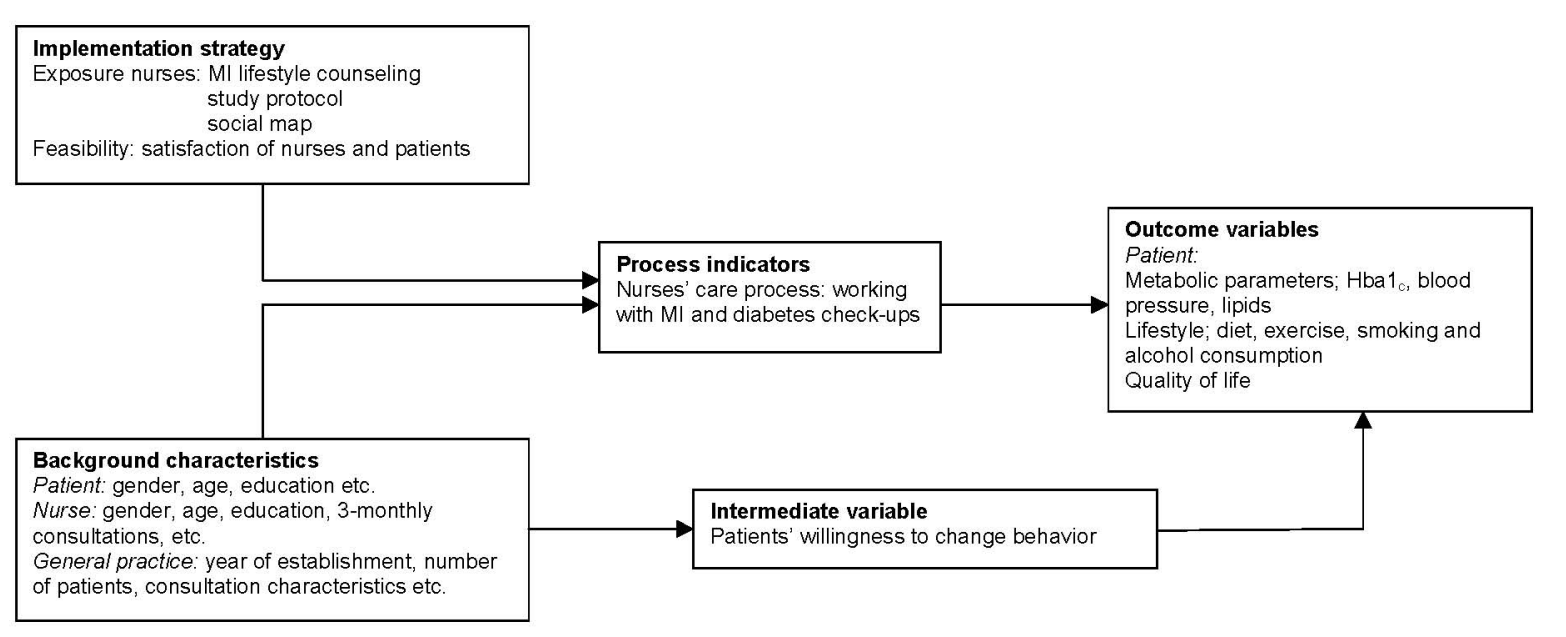
**Flowchart showing relationship of the strategy, characteristics, variables, and indicators**.

Figure 2 and Table 1 give a detailed overview of the outcome measures and instruments. The outcome measures will be the care process, the patients' metabolic parameters HbA1_c_, blood pressure, lipids [low-density lipoprotein (LDL) and total cholesterol], lifestyle (diet, exercise, smoking, and alcohol consumption), health-related quality of life, and the patients' willingness to change their behavior. Working with MI and diabetes check-ups carried out by primary care nurses will provide the two measures of the care process. Data about the degree to which the nurses work according to the MI principles will be obtained from the record keeping, and data from medical files will help us gain insight into the diabetes check-ups. The metabolic parameters will be obtained from medical files. The current state of the art in measuring lifestyle changes is a multi-method approach that combines feasible self-reporting and reasonably objective measures. The patients will also report specific behaviors relating to smoking[37], saturated fat intake[38], fruit and vegetable consumption[39], physical activity[40,41] and alcohol use[42] by means of validated self-reported questionnaires. We will use an objective measurement, the personal activity meter (PAM), with a diary for recording physical activity for 7 days. A combination of subjective and objective reporting will be chosen to get insight into the amount of exercise. A self-reported questionnaire will be used to measure the health-related quality of life[43,44] and the patients' willingness to change their behavior. This willingness to change will be determined on the basis of answers to questions about the importance and the confidence to change. Other factors that will be recorded include background characteristics about the patient (sex, education, etc.), the nurse (sex, age, etc.) and the general practice (year of establishment, consultation characteristics, etc.).

**Table 1:** Effect evaluation: outcome variables and instruments used to measure them

**Outcome variables**	**Dutch guideline norms**	**Instruments**
**Care process**		
Effects of MI	-	Record keeping
Results of diabetes check-ups	-	Medical files
		
**Metabolic parameters**		
HbA1_c_	< 7%	Medical files
Blood pressure	< 140 mm Hg	Medical files
Lipids:		
LDL	< 2.5 mmol/L	Medical files
Total cholesterol	< 4.5 mmol/L	Medical files
		
**Lifestyle**		
Diet:		
Fruit and Vegetables	2 pieces fruit (200 g or more) and 200 g vegetables or more	Validated questionnaire, 8 items [39]
Fat consumption	Total fat < 35 energy percent Saturated fat < 10 energy percent	Validated questionnaire, 35 items [38]
Physical activity	30 min for 5 days/week	Questionnaire, modified Dutch version of the CHAMPS, unvalidated, 15 items [40,41]
		Personal activity meter with diary for 7 days
		
Smoking	No smoking	Validated questionnaire, 2 items [37]
Alcohol consumption	Man: maximum 2 glasses	Validated questionnaire, 2 items [42]
	Woman: maximum 1 glass	
		
**Quality of life**	-	Validated questionnaires, EQ-5D 5 items and VAS 1 item [43,44]
		
**Patients' willingness to change behavior**	-	Questionnaire

#### Timing of measurements

Information will be collected before intervention starts (T0), as well as at the end of the trial 14 months later (T1) for the intervention and control arms:

- At T0 (baseline), the research team will collect data from medical files, such as HbA1_c _concentration, blood pressure, and lipid values. Eligible patients will receive the first questionnaire from our research team (with a invitation letter from the GP) to assess their lifestyle and quality of life. Primary care nurses will instruct participating patients to carry a personal activity meter for 1 week, and to keep a diary, noting his/her physical activity for that week.

- At T1, the second and final questionnaire, PAM and diary will be sent to the patient's home address. The primary care nurse will collect data from medical files regarding medical parameters from the most recent patient contact.

The questionnaires, personal activity meters, diaries, and recording forms for medical file research are to be sent to the research team in a postage-paid envelope after completion. All these materials will carry a unique patient number, and the patient number will be related to the involved general practice number.

#### Data analysis

Dichotomization of the patient outcomes. The HbA1_c _concentration must be less than 7%; blood pressure, less than 140 mm Hg; the LDL, less than 2.5 mmol/L; and the total cholesterol, less than 4.5 mmol/L [18]. Patients must eat two pieces of fruit and 200 g of vegetables per day, must exercise 30 minutes a day at least 5 days a week, must consume less than 10 energy percent in saturated fat, must not smoke, and must consume no alcohol or only a moderate amount[45]. Descriptive statistics will be used to summarize factors of general practice, primary care nurse and patient factors for the two study arms and to check for comparability in baseline variables between the control arm and the intervention arm. We will use logistic regression techniques accounting for two levels (patient and practice) to statistically compare the intervention and control arms. The baseline measurement will be used in the model as a co-variant. Since different staff members in one practice are involved in the treatment of the diabetes nowadays (care given at practice level), we will have to choose the level of the practice. Exploratory analyses are also planned to examine the effect of explanatory factors (e.g., sociodemographic factors) on the outcome.

### Process evaluation

The purpose of the process evaluation will be to establish the actual exposure and to investigate the feasibility of the implementation strategy. The research question is, "To what extent do primary care nurses take part in the implementation strategy, and what do the nurses and patients think of this strategy?"

#### Variables and measurements

To establish exposure, we will measure the degree of application of the training program. Each nurse will be instructed to record 8 to 10 diabetes consultations before and after the trial to determine their actual MI usage. Data about the primary care nurses' actual exposure to the other components of the implementation strategy (making a diabetes protocol and social map) will be established in the training.

At the end of the study, the nurses will receive a questionnaire about the feasibility of the implementation strategy. This questionnaire consists of questions about their experience with the implementation strategy, such as, "Do you think the MILD project would be useful to other nurses?" and "Looking back, would you participate in the MILD project again?" Patients will also be asked to fill in a questionnaire about their satisfaction with the diabetes consultations with questions such as, "What did you think of the diabetes consultations last year?" For a more detailed overview of the process measures and instruments, see Figure 2 and Table 2. This process evaluation can shed light on obstacles and facilitators that can be used for broader implementation.

**Table 2:** Process evaluation: the measures and the instruments used to determine their effects

**Measures**	**Instruments**
**Exposure**	
Lifestyle counseling with MI	8–10 Video recording, which will be scores with the checklist "BECCI" [46]
Diabetes protocol	-
Social map	-
	
**Feasibility**	
Nurses' perception and experience of the implementation strategy	Questionnaire
Patients' opinion of diabetes consultations	Questionnaire

#### Data analysis

We will explore the influences on the model introduced by differences in the primary care nurses' accurate performance of MI. Video recordings of diabetes consultations in the intervention and control arms will be scored with the existing checklist, the BECCI, to determine the extent to which the MI technique was implemented[46]. Descriptive statistics will be used to summarize the patients' opinions of the diabetes consultations and the nurses' opinions of the feasibility of the implementation strategy.

### Economic evaluation

The economic evaluation will estimate the cost-effectiveness of the MILD implementation intervention. The research question will be, "What is the incremental cost-effectiveness of our implementation strategy compared to usual care given by a primary care nurse?"

#### Measurements and variables

Cost-effectiveness analysis will be done from a healthcare perspective with a lifetime horizon because changes in glycemic control affect long-term complication risks. In a previous study, we show that adherence to the diabetes guidelines is cost-effective from a perspective of a lifetime horizon[47,48]. The costs of the implementation strategy will be included, such as costs of changes in the diabetes organization, training the professionals in MI, and extra telephone calls with the patients as well as the major patient-related cost items (number and type of appointments and treatment). The effects measured in the model evaluation will be the effects on the HbA1_c _concentration as the primary outcome measure, and on exercise levels, dietary habits, cardiovascular risk score, and alcohol consumption as process indicators.

#### Data analysis

The economic evaluation will be a cost-effectiveness study. The cost will be balanced against the effect measures in a standardized model approach. In this study, policy cost-effectiveness will also be analyzed in a Mason model that includes the following sections[49]:

1. Developmental costs of the implementation

2. Training of primary care nurses

3. Activities of health professionals and patients during the intervention period, e.g., clinic visits and telephone contacts

4. Costs of treatment, e.g., for use of health care services, drugs, specialist care, and complications.

Training and material costs will be based on real costs, and national guidelines will be used[50] to calculate the cost of professional activities. The actual number of patients in the intervention arm will be used to calculate the cost of the intervention activities per patient. The volume of the patient activities will be registered in the patient files, while patients will be asked to record activities outside the general practice in a diary. We expect these costs to be less than 20 euros per treated patient. On the basis of earlier research, we know that this amount of money is certainly cost-effective[51,52].

### Time frame

**Figure 3:**
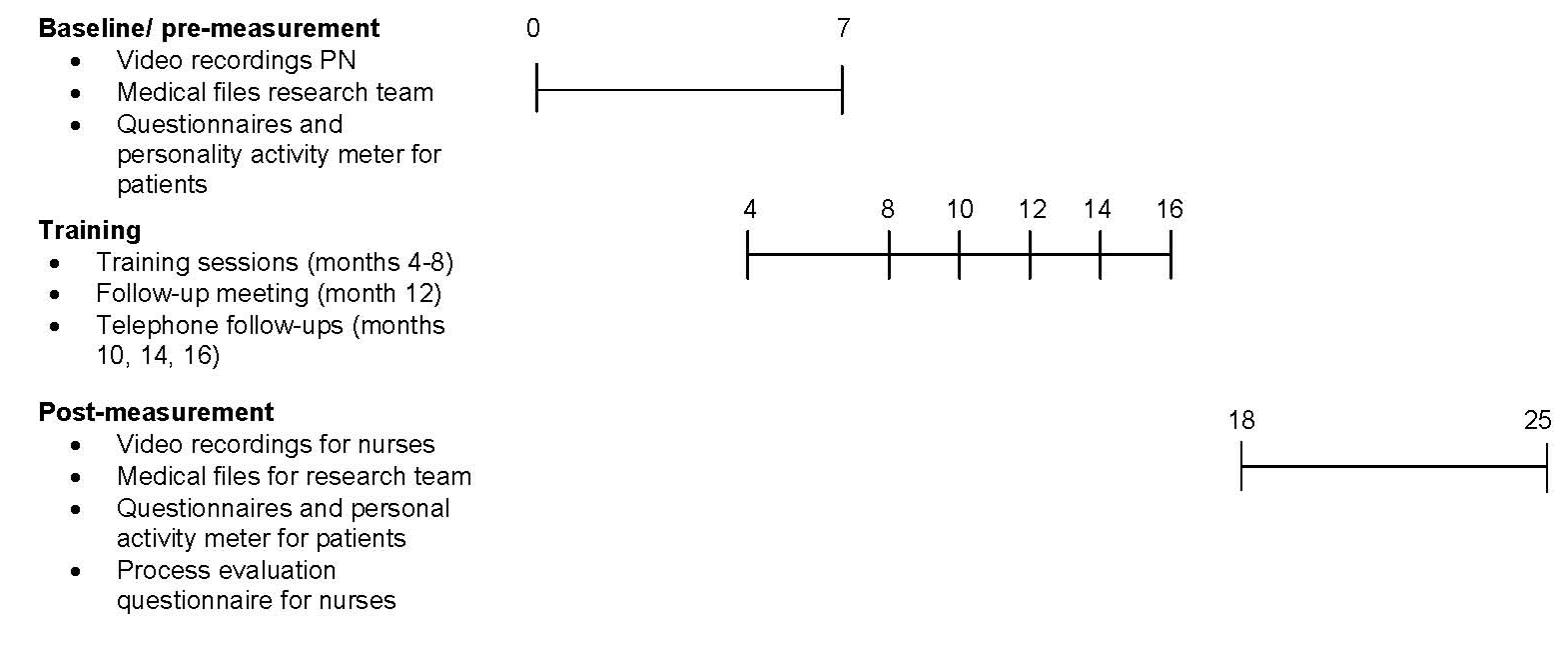
**Time frame for the Motivational Interviewing to Change the Lifestyle of Patients with Type 2 Diabetes Study**. All numbers refer to months.

We plan to randomize all general practices that declare their willingness to participate in this study. The baseline data collection will take place at the beginning of the study during the 1st 7 months (Figure 3). The training will start in month 4, and the follow-up meeting will be organized in month 12. There will be three telephone follow-ups to inquire about the nurses' development of health counseling in months 10, 14, and 16. Follow-up data collection will take place in months 18–25 inclusive. The time scheduled for the trial is 25 months.

## Discussion

The design of a cluster, randomized, controlled trial is optimal from the methodological perspective, and it could shed light on the effectiveness of the individual ingredients of this multifaceted intervention. The implementation strategy that will be evaluated in this trial is characterized by its innovative aspects. There is a wide range of interventions aimed at improving the provision of diabetes care in primary care, but not much is known yet on how to implement and integrate different intervention strategies effectively within day-to-day care in general practice[53,54]. If this intervention proves to be effective and cost-effective, its implementation will be considered and anticipated.

Selection bias is a widespread problem in cluster randomized trials[55]. Some of the biases associated with the use of cluster designs can be avoided with careful attention to the design. Identifying patients before randomizing the practices will be impossible in our study design, so we will use an independent recruiter to recruit the participants. In this way, we will take adequate precautions to guard against threats to the internal validity of the design suggested by Torgerson[56]. It is important to use a randomized design in the study because there will inevitably be other initiatives relating to diabetes that will begin during our study period, and because we will need to check for unknown effect mediators and moderators. We choose to involve many practices with relatively few patients instead of a few practices with relatively many patients for two reasons. First, more practices will increase the chance of successful implementation in more practices, and will also decrease cluster contamination. We will get also more information about the conditions necessary for implementing our strategy in a large group of practices[57,58].

We assume that getting the practice motivated for structured care will not be very difficult, if they can get some support from us for making their schedules. We also assume that it will be easy to motivate the primary care nurse to use the MI tool because an effective tool for discussing diet and exercise is currently not available in general practice. However, the nurses will have to be supported in their motivation to participate, especially in the long run. Some suggestions from the literature that we have already elaborated on in our intervention strategy are MI training followed by a follow-up meeting, an instruction chart with counseling techniques, a record keeping system for consultation data and behavioral change, and regular telephone follow-ups to the primary care nurses. These items can be helpful in maintaining motivation, as well as getting feedback about the results[59].

In contrast to these assumes, we think that it is very difficult to receive the records of the nurses in order to establish the actual usage of MI. We will provide effort to get the records of all primary care nurses, because these records are very useful.

Furthermore, we expect that there will be more effects on lifestyle outcome measures than on metabolic parameters because a lifestyle change must occur before we can measure an effect on metabolic parameters. We expect the metabolic parameters to have stronger long-term effects.

Although we originally intended to use more objective measures for the outcome measurements of the effect evaluation, logistic and financial conditions often preclude this. A biomarker for fruit and vegetables, such as carotene measurement, is expensive. There is no simple biomarker available for alcohol. The influence of information bias resulting from subjective self-reports can be reduced in the data analysis by taking the use of pre-intervention scores (which have the same information bias) into account. We will make use of practicable objective measurements of physical activity. Personal activity meters are known not to register all activities with accuracy, and the level of activity can vary from week to week, but we have chosen to combine subjective and objective data to get better insight into the amount of exercise. There are a great many different actometers in circulation. We have chosen an accelerometer, which is more accurate than the pedometer that counts the number of steps walked in a day[60,61].

## Competing interests

The authors declare that they have no competing interests.

## Authors' contributions

RJ, the main investigator, is involved in developing the intervention and the instruments, as well as in the implementation, analysis and reporting aspects of the trial. JB, the project leader, is involved in all aspects of the study. TvdW has participated in discussions about the design of the study. LN will evaluate the economics. GE advises the project team on shared decision making. RG is involved in the design of the study, the analyses and the reporting. All authors have read and approved the final version of the manuscript.

## Pre-publication history

The pre-publication history for this paper can be accessed here:



## Supplementary Material

Additional file 1 **Box 1: Implementation strategy for primary care nurses. **This box shows the various components of the implementation strategy for the primary care nurses Click here for file

Additional file 2 **Box 2: Description of motivational interviewing from Miller et al.** In this box, the motivational interviewing technique from Miller et al. is described in more detail Click here for file
