# Supplementary material for: Nurse-led motivational interviewing to change the lifestyle of patients with type 2 diabetes (MILD-project): protocol for a cluster, randomized, controlled trial on implementing lifestyle recommendations
Source: BMC Health Serv Res. 2009 Jan 30;9:19. doi: 10.1186/1472-6963-9-19 (PMC2646713; doi:10.1186/1472-6963-9-19)
Supplement: Additional file 1 — Box 1: Implementation strategy for primary care nurses. This box shows the various components of the implementation strategy for the primary care nurses [file 1472-6963-9-19-S1.doc]

**Training program** (4 half-days, spread equally over 6 months)

1. Training primary care nurses in the **principles of motivational interviewing (MI)** in order to encourage patients with diabetes to adhere to lifestyle guidelines. The following components will be discussed:

- *Agenda setting* to make consultations more structured and to draw up concrete appointments
- *Building motivation for change: importance and confidence*
- *Asking open questions, listening reflectively, affirming, summarizing, and eliciting change*
- *Expressing empathy, developing discretion, rolling with resistance, and supporting self-efficacy.*

1. Adapting the **diabetes protocol** to local circumstances.
2. Introducing a **social map** for lifestyle change to primary care nurses. The map is an overview of all available organizations and their treatment programs to help patients choose, for example, the right sport school or physiotherapist.

**Follow–up** (8 months)

1. **General practice tools** for primary care nurses for maintenance of the training program , which include:

- An *instruction chart with counseling techniques*, as a reminder to help patients change
- *Record keeping* of consultation data and behavioral change of the patients, which primary care nurses must do.
- Recommendations for *regular telephone follow-ups* for diabetes patients, which will be monthly in the 1st half year and then will probably decrease.
- A *help desk*: the research team will call the primary care nurse three times to inquire about their development of health counseling and nurses can call the research team for information.
- A *follow-up meeting* to receive feedback about their own video recording.
