# Supplementary material for: Nurse-led motivational interviewing to change the lifestyle of patients with type 2 diabetes (MILD-project): protocol for a cluster, randomized, controlled trial on implementing lifestyle recommendations
Source: BMC Health Serv Res. 2009 Jan 30;9:19. doi: 10.1186/1472-6963-9-19 (PMC2646713; doi:10.1186/1472-6963-9-19)
Supplement: Additional file 2 — Box 2: Description of motivational interviewing from Miller et al. In this box, the motivational interviewing technique from Miller et al. is described in more detail [file 1472-6963-9-19-S2.doc]

Motivational Interviewing (MI) is formally defined as a client-oriented, directive method for enhancing intrinsic motivation to change by exploring and resolving ambivalence. The MI approach is distinguished from some other counselling models; it is not focussed on 'I will change you', but on 'If you wish, I can help you change'. The four guiding principles of MI are (1) express empathy, (2) develop discrepancies, (3) roll with resistance, and (4) support self-efficacy.

1. Expressing empathy involves providing clients with an atmosphere of respect and acceptance of their position. The technique used is reflective listening and this is generally considered the foundation of MI and is recommended throughout the counselling process. Example script: 'Sounds like working on getting exercise and keeping up your blood glucose is very demanding. I think it is natural to struggle sometimes. What is it like for you? Are there any obstacles that make it particularly difficult?'
2. The second principle of MI involves creating a 'gap' between the client's current behaviour and their broader goals, thus cultivating motivation for lifestyle change. When the client recognizes such discrepancies, a certain level of discontent arises that makes change more likely to occur. Exploring the client’s important life values and reviewing how their current behaviours affect their ideal lifestyle and develop discrepancies. Example script: 'So on the one hand you are not sticking with your exercise programme, because it's hard to find time but on the other hand you think exercise would make you feel better and help manage your blood glucose level. It sounds like managing diabetes is pretty important to you. How do you think having a high BMI affect this overall? Where do the exercises fit in here?'
3. Directly challenging resistance is counterproductive to lifestyle change because it typically results in the client defending their current state of affairs. Rather, resistance should be rolled with and channelled instead of confronted. Rolling with resistance invites the client to consider a new perspective versus having it imposed. Example script: 'It can be very frustrating to make all these changes, especially when it has becoming a habit and others giving you hard time. I think it is completely normal to want to go back to old habits when times are tough. May I tell you about some different options that have been worked well for others?'
4. Self-efficacy, or one's confidence in changing a specific behaviour under difficult circumstance, should be supported whenever possible because it is one of the best predictors of treatment outcome. Self-efficacy can be strengthened by affirming past success (i.e., reinforcement), presenting success stories of others (i.e., modelling), and expressing their belief in the client's potential to change. Example script: 'I see you have been keeping up your blood glucose level despite the difficulties adhering to your diet and exercises. It looks like you had a lot of initial success when you began making health changes. What worked so well for you then? Sometimes a setback can actually be a good thing.'
